# Application of a highly simulated and adaptable training system in the laparoscopic training course for surgical residents: Experience from a high-volume teaching hospital in China

**DOI:** 10.1016/j.heliyon.2023.e13317

**Published:** 2023-02-02

**Authors:** Xueliang Zhou, Yanfei Shao, Chao Wu, Luyang Zhang, Jiayu Wang, Ruijun Pan, Jing Sun, Weiguo Hu

**Affiliations:** a Department of General Surgery, Ruijin Hospital, Shanghai Jiao Tong University School of Medicine, Shanghai, 200025, China; b Shanghai Minimally Invasive Surgery Center, Shanghai, 200025,China; c Department of Teaching and Research Section of Surgery, Ruijin Clinical Medical College, Shanghai Jiao Tong University School of Medicine, Shanghai, 200025, China; d Ruijin Clinical Medical College, Shanghai Jiao Tong University School of Medicine, Shanghai, 200025, China; e Department of Medical Simulation, Ruijin Clinical Medical College, Shanghai Jiao Tong University School of Medicine, Shanghai, 200025, China

**Keywords:** Highly simulated and adaptable, Laparoscopic training system, Laparoscopic surgery, Advanced integrated two-stage laparoscopic simulation training course, Standardized training for surgical residents

## Abstract

**Objective:**

To explore the effectiveness, feasibility, and training effect of a highly simulated and adaptable laparoscopic training system in the advanced integrated two-stage laparoscopic simulation training course for surgical residents.

**Methods:**

This study prospectively took the surgical residents who received the advanced integrated two-stage laparoscopic simulation training course in our hospital from December 2019 to December 2021 as the research objects. In the stage one course, the trainees are randomly distributed into the dry simulation system group and Darwin laparoscopic training system group. The subjective assessment results of the trainees from the two groups are collected by questionnaires, and the simulation assessment results of the two groups are evaluated in a unified, objective, and standardized assessment form. The pre-course and post-course questionnaires were used to evaluate the feasibility and effectiveness of the Darwin system in the stage two course.

**Results:**

A total of 62 trainees completed the stage one and stage two courses. In the stage one course, the trainees were randomly distributed into the dry simulation trainer group (N = 19) and the Darwin group (N = 43). The results of the subjective assessment questionnaire showed that compared with the dry simulator group, the students in the Darwin group had higher subjective scores (*P* < 0.05). The objective assessment results for the 3 modules of "One Track Transfer", "One Tunnel Pass" and "High and Low Pillars" in the Darwin group were significantly better than those in the dry simulator group (*P* < 0.05). The trainees who received the stage two course completed the questionnaires before and after the course. The results showed that compared with pre-course evaluation, "basic theoretical knowledge of laparoscopy", "basic skills of laparoscopy", "laparoscopic suture technique" and "camera-holding technique" were significantly improved after training (*P* < 0.05).

**Conclusion:**

The highly simulated and adaptable laparoscopic training system is effective and feasible in the advanced integrated two-stage laparoscopic simulation training course for surgical residents.

## 1 Introduction

Due to the rapid development of minimally invasive technology over the past several decades, laparoscopic surgery has gradually matured, and many new and advanced laparoscopic instruments and facilities have been developed and employed [1,2]. Laparoscopic surgery, as one of the most prominent minimally invasive surgical techniques in the 21st century, has been successfully applied in a wide range of surgical fields, including general surgery, thoracic surgery, urology, and gynecology, which has become a major direction in the development of surgery [3,4]. However, laparoscopic surgery is often more challenging than traditional open surgery due to the limited degree of freedom of laparoscopic instruments, their low flexibility, and their limited tactile feedback [[5], [6], [7]]. In light of this, laparoscopic skill training has become a mandatory course for most surgeons during not only their surgical training period but throughout their entire careers as well. In response to the development and standardization of continuous medical training in China, particularly the improvement of the nationally unified training system for residents, higher standards and challenges have been placed on the training of basic skills in laparoscopic surgery.

Presently, the majority of domestic training courses adopt simply dry simulators, but they are limited to beginners and do not provide authentic clinical practice [8,9]. In terms of a well-developed laparoscopic training course, in order to adapt to the rapid development of laparoscopic techniques in China, as well as to enhance the precision and practicality of simulation training, the Department of Teaching and Research Section of Surgery and the Department of Medical Simulation of the Ruijin Hospital have implemented a self-developed highly simulated and adaptable laparoscopic training system (Darwin® laparoscopic training system) into the existing training course to optimize the course settings. We evaluated the feasibility and training effects of this laparoscopic training system in an advanced integrated two-stage simulation training program for surgical residents, intending to evaluate the possibility of incorporating novel techniques into training programs for young surgeons.

## 2 Materials and methods

### 2.1 Training systems

**Dry simulation trainer:** Tianyan® Laparoscopic Training System (SUV0300002ACC, Group Laparoscopic Teaching and Training System).

**Fig. 1:**
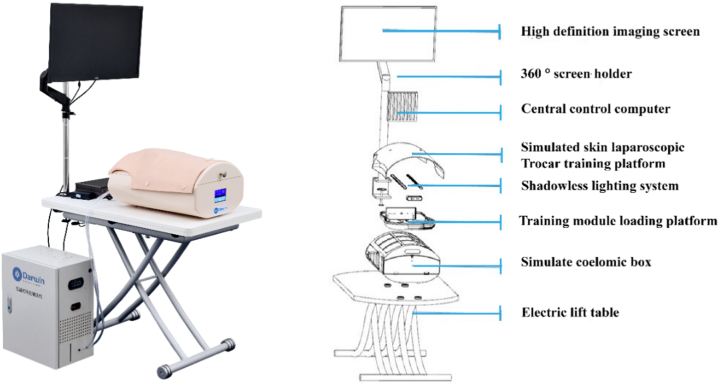
Darwin® laparoscopic training system.

**The highly simulated and adaptable laparoscopic training system:** Darwin Laparoscopic Training System (DA-102, Darwin® Multidisciplinary Minimally Invasive Skills Training System). This system is developed based on the concept of WET LAB (wet training). The integrated device system for the training of multidisciplinary minimally invasive skills has a high degree of compatibility, encompassing not only mold practice, but also independent animal specimen practice, which covers the majority of abdominal surgeries. Therefore, this system is suitable for laparoscopic basic skills as well as various advanced surgical techniques. Following the all-in-one concept, the system complements almost all the advanced laparoscopic training facilities, including electrosurgery, energy platforms, and ultrasonic scalpels, simulating real-time operations in the daily practice of surgeons (Fig. 1).

### 2.2 Curriculum design

#### 2.2.1 Theoretical learning of basic knowledge in laparoscopic surgery

The senior attending surgeons of the Department of General Surgery and the teachers of the Department of Medical Simulation will teach the history and current situation of minimally invasive surgery through theoretical lectures. Also, mentors will explain the structure, application, and proper use of basic laparoscopic instruments. The theoretical knowledge learning course is about 30min.

#### 2.2.2 Instructional video watching and debriefing

In addition to receiving theoretical knowledge training, trainees can gain a deeper understanding of the use of laparoscopic equipment and instruments by watching an instructional video. They will also gain a better understanding of the implementation process of laparoscopic systems, instruments, and basic techniques, as well as increase their enthusiasm for laparoscopic surgery. Afterward, trainees can ask questions and make comments regarding the details contained in the instructional video, as well as analyze, discuss and debrief in a group-based manner. The mentor will give a corresponding explanation and guidance. This training module is about 40 min.

#### 2.2.3 The stage one course

**Fig. 2:**
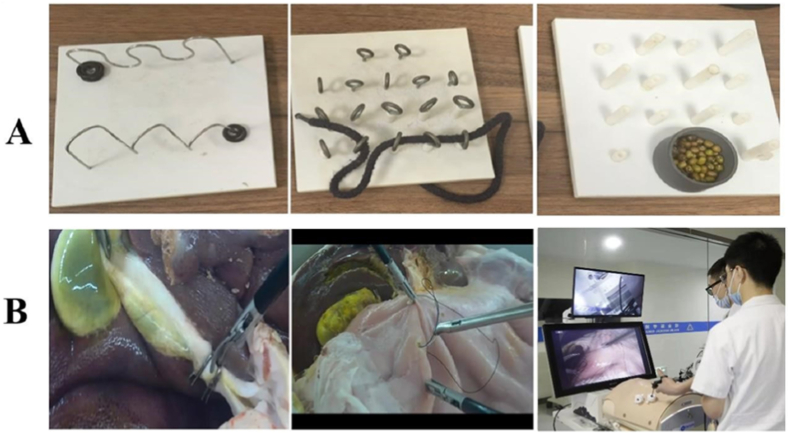
Course training molds (A: The stage one course training molds; B: The stage two course training molds).

**Fig. 3:**
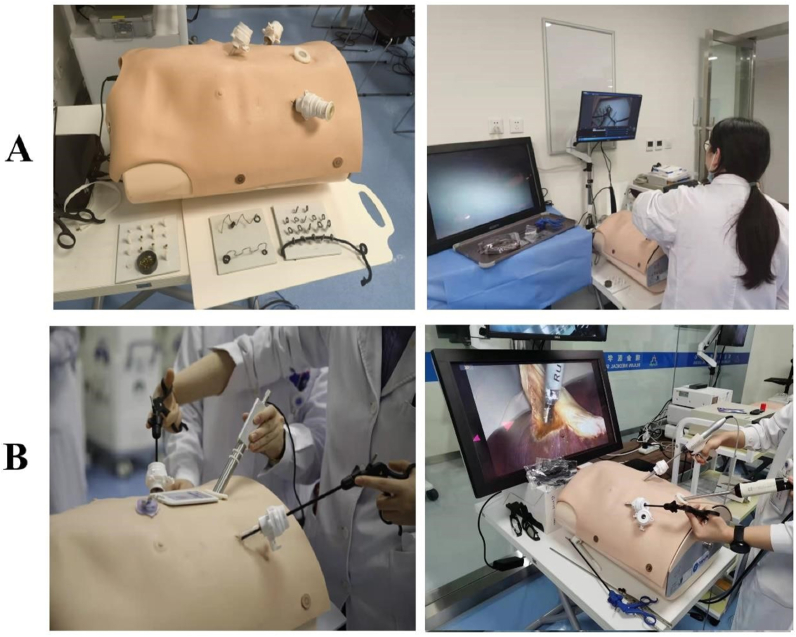
Training scenes of the advanced integrated two-stage laparoscopic simulation training course for junior surgical residents (A: The stage one course training; B: The stage two course training).

The stage one training course enables students to master the use of basic laparoscopic surgical instruments such as separation forceps and grasping forceps, as well as the basic operation skills of laparoscopic surgery such as grasping, passing, and eye-hand coordination. This program involves a simple mold pattern practice, including One Track Transfer, One Tunnel Pass, and High and Low Pillars (Fig. 2A). The training time for each module is about 20 min. The assessment method is that each resident completes One Track Transfer (one rubber ring completely goes through one track), One Tunnel Pass (one rope goes through a horizontal row of tunnels), and High and Low Pillars (beans fill all the pillars) under either the dry simulator or Darwin training system. A video recording of the entire process will be automatically generated by the built-in camera system, and the original assessment video will be saved for future reference. Mentors will review the videos to count the number of irregular movements in the process, record the completion time and give a score for the operation. Fig. 3A shows the training records of the stage one course.

#### 2.2.4 The stage two course

In the stage two course, by utilizing the highly simulated and adaptable Darwin laparoscopic training system, residents are able to develop their sense of space and touch under laparoscopy, as well as practice fine laparoscopic operation techniques, such as precise positioning, delivery, suture, and knotting under supervision. This training session is based on the independent animal abdominal multi-organ model (cholecystectomy and gastric perforation repair model, Fig. 2B), in which the training time for cholecystectomy was 20 min, and the repair time for gastric perforation was 40 min. The assessment method is as follows: 3 students in each group take turns serving as the chief surgeon, the first assistant, and the camera holder to complete the cholecystectomy and gastric perforation repair. Recording the time and degree of completion as the assessment results and keeping the original assessment videos for future reference. Fig. 3B shows the training records of the stage two course.

#### 2.2.5 Study population & study design

From December 2019 to December 2021, the first- and second-year junior surgical residents who received standardized residency training at Ruijin Hospital, Shanghai Jiao Tong University School of Medicine were prospectively enrolled as the subjects of this study and all of the enrolled residents have signed the informed consent.

Inclusion criteria for the study population: 1) residents receiving standardized residency training at Ruijin Hospital, Shanghai Jiao Tong University School of Medicine; 2) between 20 and 35 years old; 3) junior residents in the first- or second-year; 4) majoring in surgery and with a strong involvement in laparoscopic techniques in their field; 5) received and completed the theoretical and practical courses in clinical medicine as required; 6) volunteered to participate in this study and have a preliminary knowledge of laparoscopic techniques; 7) are in good physical and psychological health.

After the trainees received the theoretical courses intensively, they were 2:1 randomly divided into two groups to carry out simulation practice on Darwin laparoscopic training system and the dry simulation trainer for the stage one course. In total, 62 surgical residents have completed the subjective scoring questionnaires and a unified, objective, and standardized assessment of advanced integrated stage one laparoscopy simulation training as required, including 19 in the dry simulation trainer group and 43 in the Darwin laparoscopic training system group.

**Fig. 4:**
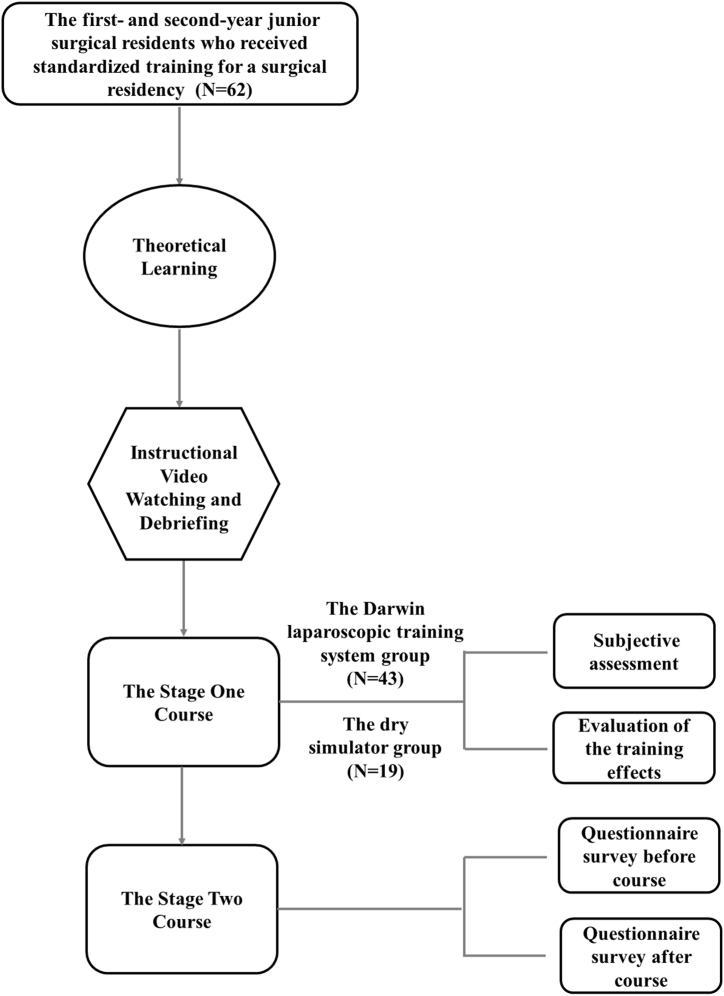
The flow chart of the study design.

For the residents who received the stage two course based on Darwin laparoscopic training system, pre- and post-course online questionnaires were collected in the trainee cohort to assess the feasibility and training effect of the Darwin laparoscopic training system in practice. In total, 62 trainees who completed the stage one course received the stage two course based on Darwin laparoscopic training system and completed the pre-and post-course questionnaires. The flow chart of the study design is shown in Fig. 4.

### 2.3 Questionnaire

The study employed a self-administered comprehensive questionnaire to collect baseline information, subjective assessment of the stage one curriculum setting and self-assessments before and after the stage two course from all study participants (Supplementary Materials). The questionnaire was conducted in the form of self-filling test, which was divided into four sections: “Baseline information collection”, “The subjective assessment of the stage one curriculum setting”, “The questionnaire survey before stage two course” and “The questionnaire survey after stage two course”.

All study participants volunteered to take part in the questionnaire and answered it on their own without any interference. After completion of the questionnaire, it is quality controlled by the appropriate professional and collected on the spot.

### 2.4 Statistical analysis

SPSS 18.0 software was used for data analysis. Enumeration data were statistically analyzed using Chi-square test. Measurement data were expressed as mean ± standard deviation, Student’s t-test was used to compare variables that conformed to a normal distribution, and Wilcoxon rank-sum tests were used to compare variables that were not normally distributed. Bilateral statistical tests were used for *P* values of all statistical data and *P* < 0.05 was considered statistically significant.

## 3 Results

### 3.1 Baseline information and distribution on the trainees between the Darwin system group and the dry simulator group

A total of 62 trainees were prospectively enrolled in this study from December 2019 to December 2021 at our hospital. After an intensive theoretical course, the trainees were randomly divided in a 2:1 ratio into Darwin laparoscopic system group (N = 43) and the dry simulator group (N = 19), with baseline information and distribution of the trainees between the two groups shown in Table 1. As seen in Table 1, there were no significant differences in "Age" (χ^2^ = 0.934, *P* = 0.704), "Gender" (χ^2^ = 0.138, *P* = 0.710), "Years of residency" (χ^2^ = 0.332, *P* = 0.564), "Interest in laparoscopic surgery" (χ^2^ = 0.498, *P* = 0.480) and "Years of laparoscopic operation" (χ^2^ = 2.350, *P* = 0.726) among the trainees in two groups. Therefore, the random distribution of the trainees in two groups is reasonable and the effects of the laparoscopic training course are well comparable between the two groups.

**Table 1:** Baseline information and distribution on the trainees between Darwin laparoscopic system group and the dry simulator group.

Items	Darwin laparoscopic system group (N = 43, %)	The dry simulator group (N = 19, %)	χ^2^	*P* value
Age (years old)				
20–25	19 (44.2)	7 (36.8)	0.934	0.704
26–30	23 (53.5)	11 (57.9)		
31–35	1 (2.3)	1 (5.3)		
**Gender**				
Male	25 (58.1)	12 (63.2)	0.138	0.710
Female	18 (41.9)	7 (36.8)		
**Years of residency (years)**				
1	26 (60.5)	10 (52.6)	0.332	0.564
2	17 (39.5)	9 (47.4)		
**Interest in laparoscopic surgery**				
None	0 (0.0)	0 (0.0)	0.498	0.480
Low	0 (0.0)	0 (0.0)		
Moderate	12 (27.9)	3 (15.8)		
High	31 (72.1)	16 (84.2)		
**Years of laparoscopic operation (years)**				
0	3 (7.0)	1 (5.3)	2.350	0.726
1	23 (53.4)	7 (36.8)		
2	12 (27.9)	8 (42.1)		
3	3 (7.0)	2 (10.5)		
More than 3	2 (4.7)	1 (5.3)		

The highly simulated and adaptable laparoscopic training system is feasible for the stage one training course.

The results of the subjective scoring questionnaires for the stage one course showed that there were statistically significant differences between the Darwin system group and the dry simulator group in terms of "classroom environment layout and atmosphere" [(4.93 ± 0.26) vs. (4.47 ± 0.70), *P* = 0.012], "adequate practice opportunities" [(4.98 ± 0.15) vs. (4.53 ± 0.61), *P* = 0.005], "course contributes to clinical work" [(4.98 ± 0.15) vs. (4.68 ± 0.48), *P* = 0.017], "course meets pre-course expectations" [(4.95 ± 0.21) vs. (4.37 ± 0.76), *P* = 0.004] and "training supplies preparation" [(4.91 ± 0.29) vs. (4.47 ± 0.51), *P* = 0.002], but there were no statistically significant differences between the two groups in terms of "teaching methods and schedule" [(4.88 ± 0.32) vs. (4.68 ± 0.58), *P* = 0.175]. The results show that compared with the dry simulator, the trainees have higher subjective scores for the Darwin training system and no obvious subjective discomfort. Therefore, it is feasible to adopt the Darwin laparoscopic training system to replace the simply dry simulator in the stage one course of advanced integrated two-stage laparoscopy simulation training (Table 2).

**Table 2:** The subjective assessment of the stage one curriculum setting.

Items	Darwin laparoscopic system group (N = 43)	The dry simulator group (N = 19)	t	*P* value
Classroom environment layout and atmosphere	4.93 ± 0.26	4.47 ± 0.70	2.77	0.012
Teaching methods and schedule	4.88 ± 0.32	4.68 ± 0.58	1.40	0.175
Adequate practice opportunities	4.98 ± 0.15	4.53 ± 0.61	3.17	0.005
Course contributes to clinical work	4.98 ± 0.15	4.68 ± 0.48	2.61	0.017
Course meets pre-course expectations	4.95 ± 0.21	4.37 ± 0.76	3.30	0.004
Training supplies preparation	4.91 ± 0.29	4.47 ± 0.51	3.44	0.002

### 3.2 Evaluation of the training effects of the Darwin laparoscopic training system group and the dry simulation trainer group

After completing the stage one training course of the advanced integrated two-stage laparoscopy simulation training, the trainees in the two groups participated in a unified, objective, and standardized assessment. The trainees completed the assessment of three modules of "One Track Transfer", "One Tunnel Pass" and "High and Low Pillars" by laparoscopy. The number of irregular movements [(2.00 ± 1.29) vs. (3.32 ± 2.21), *P* = 0.024] and score [(4.42 ± 0.50) vs. (3.74 ± 0.93), *P* = 0.006] of trainees operating the Darwin training system were significantly better than those of the dry simulator in the module of "One Track Transfer" with a statistically significant difference between the two groups, and no statistically significant difference in time [(155.09 ± 72.79) vs. (193.26 ± 96.53), *P* = 0.091] between the two groups. In the module of "One Tunnel Pass", the score [(4.21 ± 0.74) vs. (3.68 ± 0.58), *P* = 0.008] of trainees operating the Darwin training system were better than those of the dry simulator, but there was no significant difference in time [(251.49 ± 121.53) vs. (257.47 ± 124.25), *P* = 0.860] and the number of irregular movements [(2.19 ± 1.40) vs. (2.79 ± 1.81), *P* = 0.159]. In the module of "High and Low Pillars", the score [(4.07 ± 0.55) vs. (3.63 ± 0.76), *P* = 0.032] of trainees operating the Darwin training system were better than those of the dry simulator, but there was no significant difference in time [(260.19 ± 86.16) vs. (329.63 ± 190.72), *P* = 0.052] and the number of irregular movements [(4.84 ± 2.23) vs. (5.37 ± 3.70), *P* = 0.567]. Therefore, the above results indicate that the overall performance of the Darwin training system is significantly better than that of the dry simulator, and the Darwin laparoscopic training system is more advantageous than the dry simulator for the stage one training course of the advanced integrated two-stage laparoscopy simulation training, and that it is more conducive to the acquisition of basic laparoscopic skills and significantly improves the training effects (Table 3).

**Table 3:** Comparison of operation assessment results between the Darwin laparoscopic system group and the dry simulator group.

Assessment modules	Darwin laparoscopic system group (N = 43)	The dry simulator group (N = 19)	t	*P* value
One Track Transfer				
Time (seconds)	155.09 ± 72.79	193.26 ± 96.53	−1.72	0.091
Number of Irregular Movements (times)	2.00 ± 1.29	3.32 ± 2.21	−2.42	0.024
Score (points)^a^	4.42 ± 0.50	3.74 ± 0.93	3.00	0.006
**One Tunnel Pass**				
Time (seconds)	251.49 ± 121.53	257.47 ± 124.25	−0.18	0.860
Number of Irregular Movements (times)	2.19 ± 1.40	2.79 ± 1.81	−1.43	0.159
Score (points)^a^	4.21 ± 0.74	3.68 ± 0.58	2.73	0.008
**High and Low Pillars**				
Time (seconds)	260.19 ± 86.16	329.63 ± 190.72	−1.99	0.052
Number of Irregular Movements (times)	4.84 ± 2.23	5.37 ± 3.70	−0.58	0.567
Score (points)^a^	4.07 ± 0.55	3.63 ± 0.76	2.26	0.032

a Each assessment is independently scored by three senior specialists experienced in laparoscopic training from five perspectives: depth of touch, manual dexterity, the efficiency of handling, use of instruments, and problem-solving skills. The average value is taken as the final score of the operation assessment.

### 3.3 Questionnaire before and after the stage two course training

The trainees who received the stage two course of the advanced integrated two-stage laparoscopy simulation training completed pre-course and post-course questionnaires. The survey results showed that, compared with before the training course, the trainees believed that "basic theoretical knowledge of laparoscopy" [(2.95 ± 1.15) vs. (3.95 ± 0.91), *P* < 0.001], "basic skills of laparoscopy" [(2.68 ± 1.16) vs. (3.76 ± 1.04), *P* < 0.001], "laparoscopic suture technique" [(2.32 ± 1.20) vs. (3.46 ± 1.12), *P* < 0.001] and "camera-holding technique" [(3.14 ± 1.32) vs. (4.22 ± 0.95), *P* < 0.001] were significantly improved after completing the stage two course training. This further demonstrates the feasibility and effectiveness of the Darwin laparoscopic training system in the stage two course training of the advanced integrated two-stage laparoscopy simulation training, with no obvious discomfort and greater acceptance by the trainees, as a better alternative training system for simulation such as live animal experiments in the advanced clinical training course (Table 4).

**Table 4:** The results of the questionnaire survey before and after the stage two course.

Items	Training for the stage two course (N = 62)			
	Before course	After course	t	*P* value
Basic theoretical knowledge of laparoscopy	2.77 ± 1.02	4.13 ± 0.67	−6.61	0.000
Basic skills of laparoscopy	2.55 ± 0.93	3.90 ± 0.87	−5.47	0.000
Laparoscopic suture technique	2.32 ± 1.01	3.65 ± 1.05	−5.09	0.000
Camera-holding technique	3.13 ± 1.18	4.39 ± 0.62	−5.04	0.000

## 4 Discussion

Laparoscopic simulation training refers to a training method that uses various simulation or virtual solutions to acquire laparoscopic skills and operation learning [[10], [11], [12]]. At present, laparoscopic simulation training has been widely carried out in many medical institutions in western countries, which can effectively shorten the clinical learning curve, and improve the laparoscopic operation quality [[13], [14], [15], [16]]. The Society of American Gastrointestinal and Endoscopic Surgeons (SAGES) launched the FLS (Fundamentals) certification program in 2004 as a nationally accredited program, which includes theoretical teaching, practical training, and standardized assessment of laparoscopic surgery. However, the medical or legal responsibilities associated with the certification of surgeons are the responsibility of the individual hospitals in Europe, such as the renowned European IRCAD-EITS (Strasbourg) and ESI (European College of Surgeons), which offer a fee-based, combined online and offline system of laparoscopic training courses [[17], [18], [19]].

At present, there is no unified laparoscopic surgery basic training course and assessment certification program in China. It is only possible to conduct systematic laparoscopic teaching and training in high-volume medical centers with a laparoscopic basic training course system. Laparoscopic technology and training equipment have been promoted widely in China, which shifted the time for surgeons to start learning and practicing laparoscopic techniques from continuing medical education to surgical residency training and even internship [20]. However, most of the young surgeons in China still acquire the basic skills of laparoscopic surgery gradually through surgical observation and clinical operation practice, which is prone to a long learning curve, non-standard operation techniques, and higher surgical complications risks. On the other hand, clinical medicine has entered an era of standardization. Clinical guidelines based on evidence-based medicine have been developed for most common diseases, and the diagnosis and treatment of diseases are gradually becoming standardized. However, the standardization of surgical procedures is relatively underdeveloped. For a long time, there has been a lack of uniform standards for the standardization, quality control and skills training of surgical procedures worldwide. It is difficult to evaluate the quality of surgical skills training as an important part of a resident's career due to the complexity of surgical procedures and the inconsistency of training programs and skills assessment procedures across training units. The ideal assessment criteria should have the following conditions: objective and impartial, professional in content, structured and quantifiable. The Objective Structured Assessment of Technical Skills (OSATS), one of these objective skills assessments, has been used by the University of Toronto since the 1990s and is expected to play an important role in the future assessment process for surgical skills training [21].

The General Surgery Department of Ruijin Hospital, Shanghai Jiao Tong University School of Medicine, and Shanghai Minimally Invasive Surgery Center took the lead in carrying out standardized professional laparoscopic skills training nationwide in 2012. It is worth mentioning that the basic laparoscopic skills training courses led and conducted by Professor Sun, the corresponding author of this article, has been internationally accredited by the Royal College of Surgeons of England (RCS). After many years of continuous curriculum development and iteration, a series of standardized and effective laparoscopic training courses has been developed, along with more training experience and teaching accomplishments. Based on the laparoscopic training courses that have been carried out since 2012, the author believes that the contradictions between ensuring medical safety and training quality, between the huge demand for training and the relative shortage of teachers, and between developing training molds and maintaining the interest of trainees need to be solved and improved by innovations in training systems, training models and training experiences respectively. The development of the advanced integrated two-stage laparoscopy simulation training and the replacement of the traditional dry simulator with the Darwin laparoscopic training system, which is compatible with both dry and *in vitro* specimens, camera holding training, and in-team training, are expected to resolve these three contradictions. This study focuses on the feasibility and training effect of the Darwin laparoscopic training system in the advanced integrated two-stage laparoscopic simulation training course for junior residents, to lay a foundation for the promotion and application of the Darwin laparoscopic training system in the future.

A variety of training platforms are currently available for laparoscopic surgery, including dry simulation trainers, computer-based virtual trainers, wet organ trainers, and live animal experiments [[22], [23], [24]]. The training platforms used by most laparoscopic training institutions in China are mainly dry simulation trainers, including homemade "black boxes" from cardboard boxes or plastic finishing boxes, as well as various merchandised products. The dry simulation trainer creates an “abdominal space” that simulates the state of the human pneumoperitoneum. The images are always obtained through the built-in camera and/or external imaging devices. Initially, it is designed to help trainees master the use of common laparoscopic instruments, practice locating depth within a two-dimensional laparoscopic field, develop hand-eye coordination skills, and learn about the basic skills of laparoscopy. It has the advantages of easy access to equipment, similar clinical instruments and low training cost [25]. Therefore, this has led to the high popularity of dry simulators, which are widely used in the initial stages of laparoscopic skill learning.

The highly simulated and adaptable Darwin laparoscopic training system we developed is an integrated and user-friendly system for multidisciplinary minimally invasive skills training based on the WET LAB (wet training) concept, which covers all the functional devices and instruments required for training, providing a high degree of compatibility. The results of the subjective scoring questionnaires on the course curriculum showed that trainees were more satisfied with "classroom environment layout and atmosphere", "adequate practice opportunities", "the course contributes to clinical work", "Course meets pre-course expectations" and "training supplies preparation" in the Darwin laparoscopic training system group than in the dry simulator group. The objective assessment results also showed that the overall assessment results of the Darwin laparoscopic training system group were significantly better than those of the dry simulator group (*P* < 0.05). This result shows that Darwin laparoscopic training system is feasible and effective in the stage one basic course of laparoscopic training, offering greater training advantages than the dry simulator, significantly improving training effects, and could be a good complement to the dry simulator with a high degree of adaptability.

It is anticipated that all types of simulation training will eventually be incorporated into clinical surgery training in its various forms. Therefore, there should be a systematic approach to the training of surgeons based on a step-by-step progression from a basic level to a high level of difficulty, from low risk to high risk, and from basic skills to surgical coordination [26]. It is worth noting that the Darwin laparoscopic training system is built around a unique supplementary model that simulates the clinical operation scenes from the perspective of a surgeon, assistant, and camera holder through the use of a realistic model of real animal organs. This allows multiple trainees with different levels to train as a team, maintain current clinical needs and develop their skills at the same time. On the contrary, the main disadvantage of the dry simulation trainer is that the content is always basic and monotonous, and it lacks the simulation of real surgical scenarios and tissue feedback. Generally, it can only be available for single-person training, so they are no longer sufficient for an advanced laparoscopic training course. Live animal experimentation is still the most acceptable platform for laparoscopic simulation training, but laparoscopic training usually adopts medium to large animals that are closer to human anatomies, such as pigs and dogs, so it needs to be conducted in a qualified animal laboratory, equipped with clinical laparoscopic equipment, instruments and anesthesia support, including pneumoperitoneum, vital monitor, and anesthesia machines [27,28]. Therefore, the single training cost of live animal experiments is relatively high, and it cannot completely cover all stages of laparoscopic training and involves animal ethical issues and risks associated with the administration of anesthetic drugs [29].

The results of the pre-course and post-course questionnaires for trainees showed that compared with the pre-course, the residents believed that after completing the stage two training course based on the Darwin laparoscopic training system, "basic theoretical knowledge of laparoscopy", "basic skills of laparoscopy", "laparoscopic suture technique" and "camera-holding technique" were significantly improved, indicating that Darwin laparoscopic training system is a great advantage in advanced laparoscopic training courses, not only because of its high degree of simulation, which allows for realistic clinical scenarios but also because of its practicality and low training cost, which makes it available to all qualified laparoscopic teaching and training units at all levels, allowing each trainee to participate in the training process under different roles in a real pattern.

Of course, due to the limitations of research conditions, a total of 62 junior surgical residents were enrolled in this study. The role of the Darwin laparoscopic training system in laparoscopic training courses, and whether it can be extended and applied in laparoscopic skill training for surgeons and standardized residency training, needs to be further validated in more prospective, multi-center and large-scale clinical studies. The effectiveness of the Darwin laparoscopic training system in shortening the clinical learning curve, improving laparoscopic operation skills, reducing surgical complications, and ensuring patient safety has not yet been established, and this study may serve as a reference and will be validated by further long-term follow-up observations. At the same time, the application of the Darwin laparoscopic training system in the training for continuing medical education, including fellows and attendings who focus on details of laparoscopic surgery methodologies, deserves further in-depth exploration.

## 5 Conclusion

The Darwin laparoscopic training system is effective and feasible in the advanced integrated two-stage laparoscopic simulation training course for junior residents. The Darwin laparoscopic training system is highly simulated and adaptable. The Darwin laparoscopic training system is a good complement to the dry simulator in basic courses and a better option in advanced courses.

## Author contributions statement

Jing Sun, Ruijun Pan: Conceived and designed the experiments; Contributed reagents, materials, analysis tools or data.

Xueliang Zhou: Conceived and designed the experiments; Performed the experiments; Analyzed and interpreted the data; Wrote the paper.

Yanfei Shao, Chao Wu, Luyang Zhang and Jiayu Wang: Performed the experiments; Analyzed and interpreted the data.

Weiguo Hu: Contributed reagents, materials, analysis tools or data.

## Funding statement

Ruijun Pan was supported by Shanghai Municipal Health Commission Clinical Research project of health industry [201940013].

Dr. Jing Sun was supported by Shanghai Science and Technology Commission biomedical science and technology support project [19441917200].

## Data availability statement

Data included in article/supp. material/referenced in article.

## Declaration of interest’s statement

The authors declare that they have no known competing financial interests or personal relationships that could have appeared to influence the work reported in this paper.
