# Supplementary material for: Application of a highly simulated and adaptable training system in the laparoscopic training course for surgical residents: Experience from a high-volume teaching hospital in China
Source: Heliyon. 2023 Feb 2;9(2):e13317. doi: 10.1016/j.heliyon.2023.e13317 (PMC9941944; doi:10.1016/j.heliyon.2023.e13317)
Supplement: Multimedia component 1 [file mmc1.pdf]

# Application of a highly simulated and adaptable training system in the laparoscopic training course for surgical residents: experience from a high-volume teaching hospital in China

## Baseline information collection

Your name

| |
|---|
| |

Your gender

| |
|---|
| |

Your mobile phone number

| |
|---|
| |

Your email address

| |
|---|
| |

The year you began your standardized residency training

| |
|---|
| |

The department where you are now

| |
|---|
| |

Your job ID/student ID

| |
|---|
| |

Interest in laparoscopic surgery

☐ None
☐ Low
☐ Moderate
☐ High

**Years of laparoscopic operation (years)**

☐ 0

☐ 1

☐ 2

☐ 3

☐ More than 3

# The subjective assessment of the stage one curriculum setting

Classroom environment layout and atmosphere (The full score is 5 points)

Teaching methods and schedule (The full score is 5 points)

Adequate practice opportunities (The full score is 5 points)

Course contributes to clinical work (The full score is 5 points)

Course meets pre-course expectations (The full score is 5 points)

Training supplies preparation (The full score is 5 points)

# The questionnaire survey before stage two course

Basic theoretical knowledge of laparoscopy (The full score is 5 points)

Basic skills of laparoscopy (The full score is 5 points)

Laparoscopic suture technique (The full score is 5 points)

Camera-holding technique (The full score is 5 points)

# The questionnaire survey after stage two course

Basic theoretical knowledge of laparoscopy (The full score is 5 points)

Basic skills of laparoscopy (The full score is 5 points)

Laparoscopic suture technique (The full score is 5 points)

Camera-holding technique (The full score is 5 points)
